# Pharmacological and Nonpharmacological Interventions to Address Insomnia Symptoms and Poor Sleep Quality Among People With HIV: Protocol for a Scoping Review

**DOI:** 10.2196/98894

**Published:** 2026-10-14

**Authors:** Elliott R Weinstein, Meghan J Linder, Jessie Y Liu, Carol Mita, Peter R Chai

**Affiliations:** 1 Behavioral Medicine Program, Department of Psychiatry, Massachusetts General Hospital, One Bowdoin Square, Suite 900, Boston, MA, 02114, United States, 1 (617) 643-4423; 2 Department of Psychology, Harvard Medical School, Boston, MA, United States; 3 Fenway Health, Boston, MA, United States; 4 Department of Epidemiology and Biostatistics, School of Public Health, Boston University, Boston, MA, United States; 5 Department of Emergency Medicine, Brigham and Women's Hospital, Boston, MA, United States; 6 Countway Library, Harvard University, Boston, MA, United States; 7 Department of Psychosocial Oncology and Palliative Care, Dana-Farber Cancer Institute, Boston, MA, United States; 8 Koch Institute for Integrated Cancer Research, Massachusetts Institute of Technology, Cambridge, MA, United States; 9 The Broad Institute, Massachusetts Institute of Technology, Cambridge, MA, United States

**Keywords:** scoping review, insomnia, poor sleep, people with HIV, pharmacological, cognitive behavioral therapy

## Abstract

**Background:**

HIV-related research has increasingly focused on addressing long-term comorbidities affecting health-related quality of life for people with HIV. Sleep-related conditions, such as insomnia, are highly prevalent among people with HIV and are associated with a variety of health challenges, including immune dysregulation, increased mental health burden, and reduced antiretroviral therapy adherence. Although there are several evidence-based pharmacological (eg, hypnotics) and nonpharmacological interventions (eg, cognitive behavioral therapy) to treat insomnia, few have been specifically tested among people with HIV.

**Objective:**

The objective of this scoping review is to characterize the current state of the field regarding pharmacological and nonpharmacological interventions for treating insomnia symptoms and poor sleep quality among adults (aged 18 years or older) with HIV.

**Methods:**

Eligible studies are those that (1) are focused on adults with HIV (aged ≥18 years), (2) examine the effects of an intervention (pharmacological or nonpharmacological) within this population, and (3) have a primary outcome dedicated to sleep quality and/or insomnia symptoms. All published manuscripts, abstracts, conference proceedings, and currently registered studies in the clinical trials registry will be considered. The review will follow the JBI scoping review methodology and adhere to the PRISMA-ScR (Preferred Reporting Items for Systematic Reviews and Meta-Analyses extension for Scoping Reviews) reporting standards. A comprehensive literature search will be conducted by searching MEDLINE, Embase, PsycInfo, CINAHL, Web of Science Core Collection, Cochrane CENTRAL, and ProQuest Dissertations & Theses Global databases in July 2026. Two reviewers will independently screen abstracts and titles using Covidence, with a third reviewer resolving any conflicts via consensus meetings. Full-text reviews of selected studies will then be conducted to determine final study eligibility. Data from included studies will then be independently extracted using a pilot-tested extraction form that captures participant characteristics, study details, intervention design, and sleep-related outcomes. A thorough narrative review with a summary table of study characteristics and findings will be generated at the end of the scoping review process.

**Results:**

The scoping review process began in April 2026. The authors anticipate identifying relevant full-text articles by December 2026 and completing the initial review of all eligible manuscripts by January 2027. Final results are expected to be consolidated and submitted for publication in the spring or summer of 2027.

**Conclusions:**

The results of this scoping review will help to contextualize the current landscape of potentially helpful interventions to improve insomnia symptoms and poor sleep quality among people with HIV through an updated, more comprehensive review of the literature. Understanding the types of pharmacological and nonpharmacological interventions to treat insomnia symptoms and poor sleep that have already been tested among people with HIV may provide targeted guidance for optimizing personalized behavioral and medical treatments to improve sleep health in this underserved population.

## Introduction

Medical advancements in antiretroviral therapy (ART) have transformed HIV from a fatal disease to a manageable chronic illness. HIV’s global incidence has decreased by almost 40% since 2010, and HIV- and AIDS-related deaths have decreased by almost 54% [1]. Additionally, with improvements along the HIV care continuum, HIV incidence is decreasing while HIV prevalence is on the rise [2]. As of 2024, the estimated number of people with HIV globally has increased to 40 million [3], predominantly because people with HIV are living longer and healthier lives. People with HIV are now expected to have life expectancies similar to those of their HIV-negative peers, representing a substantial increase from the estimated 2-year life expectancy following an HIV diagnosis in the 1980s [4]. Although the greatest global burden of HIV is concentrated in sub-Saharan Africa [3,5], the United States and other high-income countries continue to experience a persistent HIV epidemic because of intersecting epidemics of mental health conditions, stigma, and substance use among populations such as men who have sex with men, people who use injection drugs, and sex workers [6].

With HIV’s transition to a chronic condition, the focus of HIV-related research and care has gradually shifted from one primarily focused on reducing mortality to one that is concerned with addressing comorbid physical and psychological conditions experienced by people with HIV. One area that is particularly important to consider when exploring health-related quality of life among people with HIV is sleep. Compared with their age-matched peers, people with HIV are disproportionately burdened by sleep-related disorders [7] and show a higher prevalence of poor sleep quality, with estimates ranging from 24.1% to 100% compared with 10% to 40% in the general population [8]. The most common sleep-related disorders reported by people with HIV are insomnia and obstructive sleep apnea (OSA), with rates reaching as high as 35% for insomnia [9] and up to 75% for OSA [10], depending on the sample and setting. Chronic sleep disruption itself has been associated with greater depressive symptoms, anxiety, and substance use [11-13]; increased inflammatory responses [14]; and increased cardiovascular disease risk [15-19]. Among people with HIV, sleep disturbances have been associated with increased mental health burden [8,20], higher incidence of antiretroviral nonadherence [8,21], and the presence of other medical comorbidities that diminish quality of life [22]. The sequelae of health conditions resulting from poor sleep among people with HIV are uniquely challenging given the bidirectional impact between sleep disturbances and HIV disease severity. For example, sleep has a strong influence on one’s virologic control, which in turn may compromise the efficacy of ART in fully suppressing HIV [23,24]. Conversely, certain ART medications use may impact sleep architecture and lead to increased sleep fragmentation among people with HIV [22].

Sleep disturbances among people with HIV are multifactorial and should be examined within a biopsychosocial framework [25,26]. At the biological level, the HIV retrovirus impacts the immune system in several ways. When left untreated, HIV depletes an individual’s CD4+ T lymphocytes, significantly hampering a person’s ability to fight off infection [22]. Over time, chronic immune activation driven by persistent proinflammatory markers continues to alter immune functioning within both virally suppressed and unsuppressed people with HIV [27]. Although the direct pathophysiological basis of sleep and HIV is not fully understood, poor sleep itself has been implicated in the dysregulation of inflammatory and antiviral responses, which may further impact overall immune functioning among people with HIV [22,28]. Psychologically, people with HIV experience elevated levels of depression, anxiety, stigma-related stress, and chronic pain, all of which may be associated with reduced sleep quality and diminished quality of life [29-32]. Psychological comorbidity has been found to be a determinant of insomnia among people with HIV [33]. Finally, social factors such as economic marginalization, housing insecurity, and limited access to health care further contribute to sleep disturbances among people with HIV [34]. Importantly, these factors do not operate in isolation but compound one another and create a negative reinforcing cycle that further perpetuates sleep dysregulation [26,32,34].

Fortunately, there are several evidence-based treatments to reduce insomnia symptoms and improve overall sleep quality. Cognitive behavioral therapy for insomnia (CBT-I) is the frontline nonpharmacological intervention for treating insomnia [35] because of its direct effects on sleep onset latency, wake time after sleep onset, and sleep efficiency [36,37]. Additional interventions include mindfulness and relaxation-based interventions, exercise and lifestyle interventions, and chronotherapy, which uses light exposure to regulate the body’s natural circadian rhythm [37,38]. Mind-body interventions for insomnia and sleep disorders may include yoga, acupuncture, aromatherapy, meditation, and siloed sleep hygiene education or stimulus control procedures [37,38]. Although these stand-alone interventions may help to reduce insomnia symptoms, the evidence indicates that the strongest improvements occur when these interventions are combined with a comprehensive CBT-I approach [37].

In addition to nonpharmacological interventions for treating insomnia, there are several pharmacotherapy approaches also used to improve sleep. Medications such as benzodiazepines, nonbenzodiazepine hypnotic medications, orexin receptor antagonists, melatonin receptor agonists, certain antidepressants, and antihistamines [37,38] are commonly used to improve sleep among people with acute or chronic insomnia [37]. These medications primarily function by shortening sleep latency and increasing the overall duration of time asleep; however, long-term use is discouraged due to concerns regarding adverse effects [37]. Although research suggests that pharmacological interventions for insomnia are as effective as CBT-I for improving insomnia in the short term, patients who receive CBT-I demonstrate more robust long-term sleep benefits compared with those who received only pharmacological treatment [39,40].

Despite ample literature highlighting the efficacy of both nonpharmacological and pharmacological approaches to treating insomnia and poor sleep quality in the general population, little is known about sleep-focused interventions designed for and tested among people with HIV. Although several randomized controlled trials have evaluated tailored behavioral or pharmacologic sleep interventions among people with HIV, most have targeted downstream comorbidities, rather than directly addressing sleep disorders as primary treatment targets [41]. This is particularly concerning given the elevated rates of insomnia and sleep disturbances among people with HIV and the urgent need for more personalized precision medicine approaches to treating insomnia in this more medically complex population. Furthermore, although there have been a couple of reviews [24,42] examining interventions to improve sleep among people with HIV, they likely miss potentially relevant work due to their narrow scope. This scoping review aims to address the limitations of the prior systematic reviews by including research that (1) explores both pharmacological and nonpharmacological interventions to improve sleep among people with HIV; (2) reflects the experiences of people with HIV with medical comorbidities, including OSA, due to the high comorbidity of not only HIV and other medical conditions but also insomnia and OSA; (3) is published in any language (not just English or Chinese); and (4) can be found in a wider array of indexed databases and gray literature sources. In addition, this study includes data published between 2022 and July 2026 to capture more recent evidence that would not have been included in prior reviews [24,42]. As such, these gaps in the larger literature provide a strong rationale for our scoping review of both pharmacological and nonpharmacological interventions for treating insomnia symptoms and poor sleep quality among people with HIV across the life course.

## Methods

The scoping review will follow the JBI scoping review methodology, as described by Peters et al [43], and adhere to the PRISMA-ScR (Preferred Reporting Items for Systematic Reviews and Meta-Analyses extension for Scoping Reviews) reporting standards [44]. This protocol is also registered in OSF Registries (UHGC7).

### Identifying the Research Question

The primary aim of the proposed scoping review is to characterize the current state of the field regarding pharmacological and nonpharmacological interventions to address insomnia symptoms among people with HIV. Specifically, the authors will aim to answer the following question: “Based on the currently published literature, what pharmacological and nonpharmacological approaches have been tested to treat insomnia symptoms and poor sleep quality among adults aged 18 years or older with HIV?”

### Eligibility Criteria (Inclusion and Exclusion Criteria)

Eligible studies will be those that (1) study a population of adults (aged ≥18 years) with HIV, (2) assess insomnia symptoms or poor sleep quality as a primary study outcome, and (3) explore the efficacy of a pharmacological or nonpharmacological intervention for insomnia symptoms or poor sleep quality. Studies of any date, duration, design, or setting will be included if they meet the study eligibility criteria mentioned above.

Studies will be excluded if they (1) are not published in a scholarly journal, are not reported in conference proceedings or as published abstracts, do not appear in Cochrane CENTRAL or on a clinical trial registry, do not appear in ProQuest Dissertations & Theses Global, or do not appear in applicable gray literature sources (eg, relevant reports, guidelines, unpublished studies, and other documents) that may not be indexed in peer-reviewed databases; (2) are not focused on adult populations aged 18 years or older with HIV; or (3) fail to examine insomnia or sleep quality as a primary outcome.

### Identifying Relevant Studies

Studies meeting eligibility criteria will be identified by searching MEDLINE (Ovid), Embase (Elsevier), PsycInfo (American Psychological Association; EBSCOhost), CINAHL Complete (EBSCOhost), Web of Science Core Collection (Clarivate), Cochrane CENTRAL (Cochrane Library), and ProQuest Dissertations & Theses Global (ProQuest). Controlled subject terms (ie, MeSH, Emtree, APA Thesaurus of Psychological Index Terms, and CINAHL Subject Headings) will be included when available and appropriate. The search strategy will be designed and carried out by a health sciences librarian (CM). The complete search strategies for each database are provided in Multimedia Appendix 1.

A systematic gray literature search will also be conducted in parallel with the electronic database search to minimize publication bias and ensure a comprehensive review. Gray literature sources will be searched to identify relevant reports, guidelines, conference abstracts, unpublished studies, and other documents that may not be indexed in peer-reviewed databases.

The following gray literature sources will be systematically searched: government and public health agency websites (Centers for Disease Control and Prevention, National Institutes of Health, World Health Organization, Joint United Nations Programme on HIV/AIDS, and Health Resources and Services Administration), professional and clinical organizations (American Academy of Sleep Medicine, American Psychological Association, Society of Behavioral Medicine, and International Association of Providers of AIDS Care), conference abstracts (Conference on Retroviruses and Opportunistic Infections, International AIDS Society, SLEEP Annual Meeting of the Associated Professional Sleep Societies, and Society of Behavioral Medicine Annual Meeting), and Google Scholar and Google will be searched using targeted search strings, with results reviewed through the first 10 pages (approximately 100 records) per search.

Gray literature sources will be searched using a condensed version of the database search strategy and will be updated as needed to suit each source’s functionality.

### Study Screening and Selection

Between April and June 2026, the first 3 authors (ERW, MJL, and JYL) carried out a pilot screening of a draft search in MEDLINE, using Covidence systematic review software (Veritas Health Innovation), to help refine the team’s process for determining eligibility based on titles and abstracts and to identify additional search vocabulary. Following this, the librarian (CM) ran the searches in all databases and imported records into Covidence, where duplicates were removed. The 2 primary reviewers (MJL and JYL) will independently screen titles and abstracts. Consensus meetings led by the first author (ERW) will resolve any conflicts that arise during the screening process.

After all abstracts and titles are screened, the 2 original reviewers (MJL and JYL) will obtain the full texts of these selected studies with assistance from the first author (ERW) as needed. Any identified abstracts and full texts not in English will be translated using DeepL SE, an AI translation service that has been recommended for use in scoping reviews by members of the JBI [45]. Then, to check DeepL’s translations for accuracy, we will leverage the authors’ professional networks to identify members of our community who may speak the language of the non-English records. This will serve as a check on DeepL’s translations. If no one within the authors’ professional networks speaks or is willing to translate the identified language, Google Translate will be used as a secondary check. Each reviewer will independently screen the full manuscripts of potentially eligible studies to determine final eligibility for the scoping review based on the inclusion criteria. Should any manuscript, clinical trial registry, or gray literature source fail to include required information to determine scoping review eligibility, the first author (ERW) will reach out to manuscript or trial authors via email, if possible, to elicit the necessary content. The same process for addressing any disagreements through consensus meetings will also be used during this stage of the review. The final study screening and selection process will be documented in the final scoping review via a PRISMA (Preferred Reporting Items for Systematic Reviews and Meta-Analyses) flowchart.

### Charting the Data and Data Extraction

After the screening process is complete, relevant data from included manuscripts will be entered into a shared Google Sheets data extraction tool. The same 2 main reviewers (MJL and JYL) will independently complete the data extraction process using an extraction form designed by ERW and PRC. The data extraction process outlined in this protocol paper will be pilot-tested on the first 5 articles, and any necessary adjustments to the approach will be made after another team consensus meeting consisting of ERW, MJL, and JYL. Once the data extraction process is optimized, the 2 main reviewers (MJL and JYL) will continue through the included articles using the agreed-upon methodology. Data domains to be extracted will include publication details, participant information, study design, intervention design, sleep-related outcomes, other related outcomes, and key findings and limitations. Multimedia Appendix 2 outlines the various data that will be extracted from relevant texts.

### Collating, Summarizing, and Reporting the Results

Results from the data extraction tool will be combined and synthesized to provide a comprehensive narrative review of the published literature on pharmacological and nonpharmacological interventions to address insomnia symptoms and poor sleep quality among people with HIV. A complementary table summarizing study characteristics (eg, authors, year of publication, country of origin, study design, sample size, evaluated sleep-related outcomes, a short description of the target intervention, and a review of findings) for each included manuscript will also be provided in the final scoping review.

### Patient and Public Involvement

Patients and the public were not and will not be involved in the development, design, or implementation of this scoping review.

### Ethical Considerations

As this is a review of previously published literature, ethical approval from the institutional review board is not required.

## Results

As of July 10, 2026, a total of 4270 potentially eligible unique titles and abstracts have been identified by a search of the predefined databases and registries (eg, MEDLINE and Embase) and imported into Covidence (refer to Figure 1 for the proposed PRISMA flowchart). Authors anticipate that relevant full texts will be identified by December 2026, and all eligible manuscripts will be initially reviewed by January 2027. Final results are expected to be consolidated and submitted for publication in the spring or summer of 2027. Although no specific funding was used for study procedures, some author time (ERW) was supported by the National Institute of Allergy and Infectious Diseases (T32AI007433).

**Figure 1.**
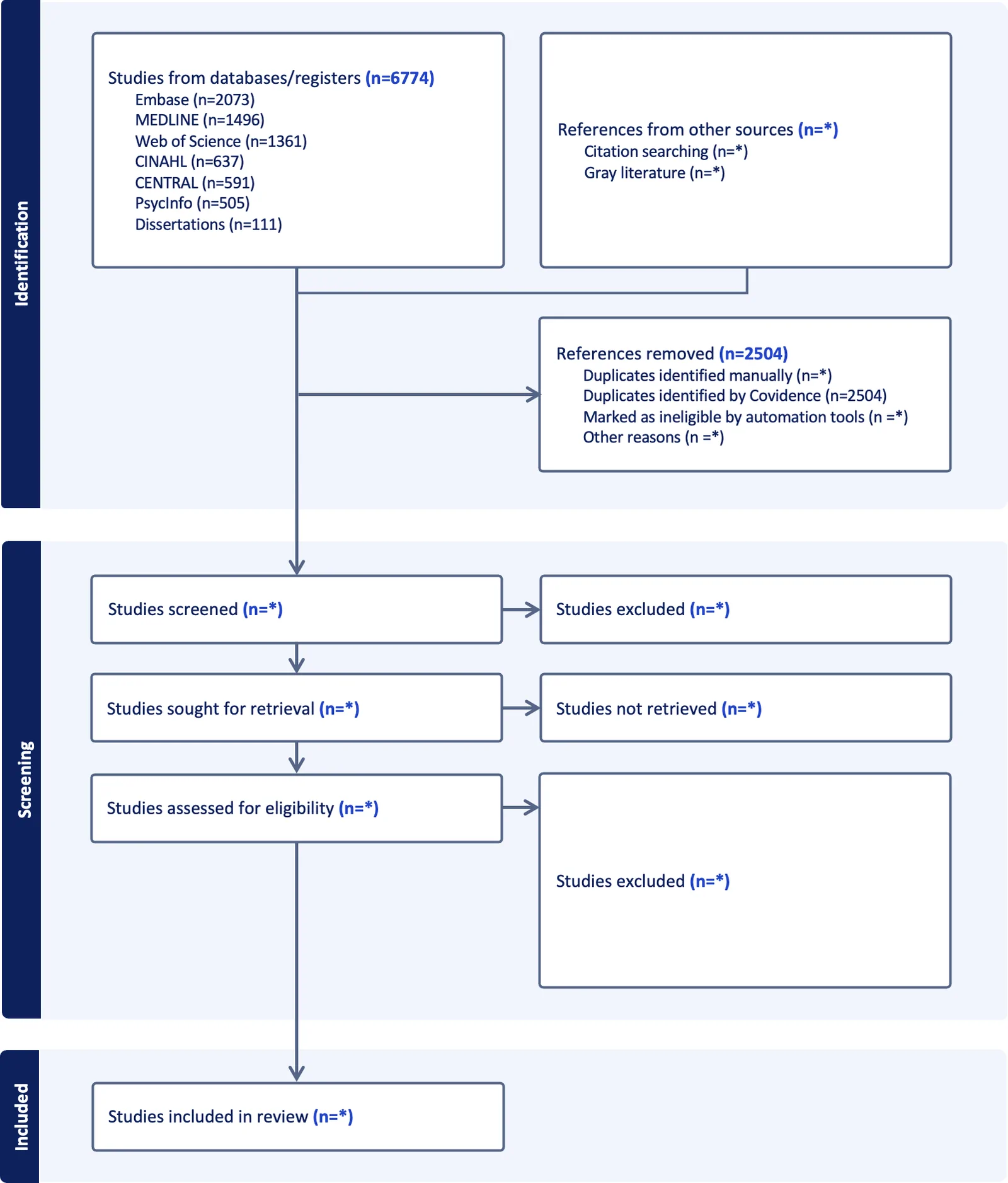
PRISMA (Preferred Reporting Items for Systematic Reviews and Meta-Analyses) flow diagram of selected studies as of July 2026 (*denotes pending steps).

## Discussion

This scoping review will provide an overview of the evidence-based pharmacological and nonpharmacological interventions that exist to address insomnia and sleep-related disorders among people with HIV. Current reviews of sleep interventions targeting people with HIV are limited and often do not include sleep as a primary outcome in interventions, solely focus on nonpharmacological interventions to treat insomnia symptoms and poor sleep, or primarily focus on studies published in English [24,42,46,47]. By identifying both currently available pharmacological and nonpharmacological insomnia treatments that have been developed and tested among people with HIV and the interventions that are most effective for this population, we may help behavioral health care clinicians and/or sleep medicine physicians deliver insomnia treatment consistent with precision behavioral medicine. This approach involves individualized assessment and personalized sequencing of behavioral treatment modules based on patients’ unique needs and may improve the effectiveness of pharmacological and nonpharmacological interventions for reducing sleep-related morbidity among people with HIV. By centering sleep as a primary clinical concern, this review may encourage more integrated, multidisciplinary strategies to address sleep disturbances directly rather than as a secondary consideration. Overall, we hypothesize that (1) few studies will be identified that specifically outline either pharmacological or nonpharmacological interventions that have been tested among people with HIV and (2) should such studies be identified, most will center on applications of CBT-I or common hypnotics and selective serotonin reuptake inhibitors most typically used to treat insomnia.

This scoping review has several anticipated limitations. Heterogeneity in study design, intervention type, and outcome measures may limit the ability to standardize comparisons across studies. These nuances may limit the strength and generalizability of the conclusions drawn from this scoping review. Additionally, variability in how sleep outcomes are defined and measured, particularly with regard to self-reported metrics, may introduce inconsistencies in the interpretation of findings. Finally, because sleep is so often assessed as a secondary outcome in HIV-related interventions, relevant studies may vary in the depth and scope with which sleep outcomes are reported and discussed.

## Supplementary material

10.2196/98894 Multimedia Appendix 1 Search terms for scoping review on pharmacological and nonpharmacological interventions to improve insomnia among people with HIV.

10.2196/98894 Multimedia Appendix 2 Domains and subdomains reflected in data extraction tool for scoping review examining pharmacological and nonpharmacological interventions to improve insomnia symptoms among people with HIV.

## Abbreviations

ART: antiretroviral therapy
CBT-I: cognitive behavioral therapy for insomnia
OSA: obstructive sleep apnea
PRISMA: Preferred Reporting Items for Systematic Reviews and Meta-Analyses
PRISMA-ScR: Preferred Reporting Items for Systematic Reviews and Meta-Analyses extension for Scoping Reviews
