# Supplementary material for: Pharmacological and Nonpharmacological Interventions to Address Insomnia Symptoms and Poor Sleep Quality Among People With HIV: Protocol for a Scoping Review
Source: JMIR Res Protoc. 2026 Oct 14;15:e98894. doi: 10.2196/98894 (PMC13664994; doi:10.2196/98894)
Supplement: Supplementary file 1 [file resprot-v15-e98894-s001.docx]

**Supplementary Appendix 1: Search Terms for Scoping Review on Pharmacological and Non-Pharmacological Interventions to Improve Insomnia among People with HIV**

*Searches*

**MEDLINE (National Library of Medicine, Ovid)**

1496 records - July 10, 2026

***Ovid MEDLINE(R) and Epub Ahead of Print, In-Process, In-Data-Review & Other Non-Indexed Citations and Daily 1946 to July 09, 2026***

1

"Sleep Wake Disorders"/ OR "Sleep Initiation and Maintenance Disorders"/ OR exp "Sleep Hygiene"/ OR (insomnia? OR ((difficult* OR problem? OR trouble?) adj4 (sleep* OR asleep)) OR (disorder* adj4 sleep) OR (disturbance? adj2 sleep) OR (disrupt* adj sleep) OR (early adj2 (awakening OR waking)) OR ((waking OR awakening) adj4 night*) OR (fragment* adj sleep) OR poor* sleep OR sleep complaint? OR sleeplessness OR nightmare? OR sleep initiation OR (quality adj2 sleep) OR sleep hygiene OR sleep restriction OR sleep education).ti,ab,kf.

2

"HIV Infections"/ OR "Acquired Immunodeficiency Syndrome"/ OR (human immunodeficiency virus OR HIV OR acquired immune deficiency syndrome OR acquired immunodeficiency syndrome OR acquired immuno-deficiency syndrome OR AIDS).ti,ab,kf.

1 AND 2

***Embase (Elsevier, embase.com)***

2073 records - July 10, 2026

Search: Advanced

Remove Embase mapping options

#1

'sleep disorder'/de OR 'insomnia'/exp OR 'sleep hygiene'/de OR 'sleep quality'/de OR (insomnia$ OR ((difficult* OR problem$ OR trouble$) NEAR/4 (sleep* OR asleep)) OR (disorder* NEAR/4 sleep) OR (disturbance$ NEAR/2 sleep) OR (disrupt* NEAR/1 sleep) OR (early NEAR/2 (awakening OR waking)) OR ((waking OR awakening) NEAR/4 night*) OR (fragment* NEAR/1 sleep) OR "poor* sleep" OR "sleep complaint$" OR sleeplessness OR nightmare$ OR "sleep initiation" OR (quality NEAR/2 sleep) OR "sleep hygiene" OR "sleep restriction" OR "sleep education"):ti,ab,kw

#2

'Human immunodeficiency virus infection'/de OR 'acute HIV infection'/de OR 'Human immunodeficiency virus 1 infection'/de OR 'Human immunodeficiency virus 2 infection'/de OR 'acquired immune deficiency syndrome'/de OR ("human immunodeficiency virus" OR HIV OR "acquired immune deficiency syndrome" OR "acquired immunodeficiency syndrome" OR "acquired immuno-deficiency syndrome" OR AIDS):ti,ab,kw

#1 AND #2 AND ([embase]/lim NOT ([embase]/lim AND [medline]/lim) OR [preprint]/lim) NOT 'tombstone'/it

***PsycInfo (American Psychological Association, EBSCOhost)***

505 records – July 10, 2026

Advanced search

Filters:

Publication Type = All journals; Dissertation Abstract

Document Type = Journal Article; Dissertation

S1

DE "Sleep Wake Disorders" OR DE "Insomnia" OR DE "Sleep Quality" OR XB (insomnia# OR ((difficult* OR problem# OR trouble#) N3 (sleep* OR asleep)) OR (disorder* N3 sleep) OR (disturbance# N1 sleep) OR (disrupt* N0 sleep) OR (early N1 (awakening OR waking)) OR ((waking OR awakening) N3 night*) OR (fragment* N0 sleep) OR "poor* sleep" OR "sleep complaint#" OR sleeplessness OR nightmare# OR "sleep initiation" OR (quality N1 sleep) OR "sleep hygiene" OR "sleep restriction" OR "sleep education") OR KW (insomnia# OR ((difficult* OR problem# OR trouble#) N3 (sleep* OR asleep)) OR (disorder* N3 sleep) OR (disturbance# N1 sleep) OR (disrupt* N0 sleep) OR (early N1 (awakening OR waking)) OR ((waking OR awakening) N3 night*) OR (fragment* N0 sleep) OR "poor* sleep" OR "sleep complaint#" OR sleeplessness OR nightmare# OR "sleep initiation" OR (quality N1 sleep) OR "sleep hygiene" OR "sleep restriction" OR "sleep education")

S2

DE "HIV" OR DE "AIDS" OR XB ("human immunodeficiency virus" OR "HIV" OR "acquired immune deficiency syndrome" OR "acquired immunodeficiency syndrome" OR "acquired immuno-deficiency syndrome" OR "AIDS") OR KW ("human immunodeficiency virus" OR "HIV" OR "acquired immune deficiency syndrome" OR "acquired immunodeficiency syndrome" OR "acquired immuno-deficiency syndrome" OR "AIDS")

S1 AND S2

***Cumulative Index to Nursing and Allied Health Literature (CINAHL Complete, EBSCOhost)***

637 records – July 10, 2026

Advanced search

Filters:

Publication Types – Doctoral Dissertation; Journal Article; Masters Thesis; Proceedings

S1

MH "Sleep Disorders" OR MH "Insomnia" OR MH "Sleep Hygiene+" OR XB (insomnia# OR ((difficult* OR problem# OR trouble#) N3 (sleep* OR asleep)) OR (disorder* N3 sleep) OR (disturbance# N1 sleep) OR (disrupt* N0 sleep) OR (early N1 (awakening OR waking)) OR ((waking OR awakening) N3 night*) OR (fragment* N0 sleep) OR "poor* sleep" OR "sleep complaint#" OR sleeplessness OR nightmare# OR "sleep initiation" OR (quality N1 sleep) OR "sleep hygiene" OR "sleep restriction" OR "sleep education")

S2

MH "HIV Infections" OR MH "HIV-Positive Persons" OR MH "Acquired Immunodeficiency Syndrome" OR XB ("human immunodeficiency virus" OR "HIV" OR "acquired immune deficiency syndrome" OR "acquired immunodeficiency syndrome" OR "acquired immuno-deficiency syndrome" OR "AIDS")

S1 AND S2

***Web of Science Core Collection (Clarivate)***

1,351 records – July 10, 2026

All Editions

Advanced Search

QUERY BUILDER

More options – Exact search

#1

TI=(insomnia$ OR ((difficult* OR problem$ OR trouble$) NEAR/3 (sleep* OR asleep)) OR (disorder* NEAR/3 sleep) OR (disturbance$ NEAR/1 sleep) OR (disrupt* NEAR/0 sleep) OR (early NEAR/1 (awakening OR waking)) OR ((waking OR awakening) NEAR/3 night*) OR (fragment* NEAR/0 sleep) OR "poor* sleep" OR "sleep complaint$" OR sleeplessness OR nightmare$ OR "sleep initiation" OR (quality NEAR/1 sleep) OR "sleep hygiene" OR "sleep restriction" OR "sleep education")

#2

AB=(insomnia$ OR ((difficult* OR problem$ OR trouble$) NEAR/3 (sleep* OR asleep)) OR (disorder* NEAR/3 sleep) OR (disturbance$ NEAR/1 sleep) OR (disrupt* NEAR/0 sleep) OR (early NEAR/1 (awakening OR waking)) OR ((waking OR awakening) NEAR/3 night*) OR (fragment* NEAR/0 sleep) OR "poor* sleep" OR "sleep complaint$" OR sleeplessness OR nightmare$ OR "sleep initiation" OR (quality NEAR/1 sleep) OR "sleep hygiene" OR "sleep restriction" OR "sleep education")

#3

AK=(insomnia$ OR ((difficult* OR problem$ OR trouble$) NEAR/3 (sleep* OR asleep)) OR (disorder* NEAR/3 sleep) OR (disturbance$ NEAR/1 sleep) OR (disrupt* NEAR/0 sleep) OR (early NEAR/1 (awakening OR waking)) OR ((waking OR awakening) NEAR/3 night*) OR (fragment* NEAR/0 sleep) OR "poor* sleep" OR "sleep complaint$" OR sleeplessness OR nightmare$ OR "sleep initiation" OR (quality NEAR/1 sleep) OR "sleep hygiene" OR "sleep restriction" OR "sleep education")

#4

TI=("human immunodeficiency virus" OR HIV OR "acquired immune deficiency syndrome" OR "acquired immunodeficiency syndrome" OR "acquired immuno-deficiency syndrome" OR AIDS)

#5

AB=("human immunodeficiency virus" OR HIV OR "acquired immune deficiency syndrome" OR "acquired immunodeficiency syndrome" OR "acquired immuno-deficiency syndrome" OR AIDS)

#6

AK=("human immunodeficiency virus" OR HIV OR "acquired immune deficiency syndrome" OR "acquired immunodeficiency syndrome" OR "acquired immuno-deficiency syndrome" OR AIDS)

(#1 OR #2 OR #3) AND (#4 OR #5 OR #6)

Refine Results:

Exclude Document Types: Editorial Material, Book Chapters, Letter, News Item, Retracted Publication

***Cochrane Central Register of Controlled Trials (Cochrane Library, Wiley)***

591 records – July 10, 2026

Search manager

Limits: turn off "Search word variations"

#1

(insomnia? OR ((difficult* OR problem? OR trouble?) NEAR/3 (sleep* OR asleep)) OR (disorder* NEAR/3 sleep) OR (disturbance? NEAR/1 sleep) OR (disrupt* NEAR/0 sleep) OR (early NEAR/1 (awakening OR waking)) OR ((waking OR awakening) NEAR/3 night*) OR (fragment* NEAR/0 sleep) OR (poor* NEXT sleep) OR (sleep NEXT complaint?) OR sleeplessness OR nightmare? OR "sleep initiation" OR (quality NEAR/1 sleep) OR "sleep hygiene" OR "sleep restriction" OR "sleep education"):ti,ab,kw

#2

("human immunodeficiency virus" OR HIV OR "acquired immune deficiency syndrome" OR "acquired immunodeficiency syndrome" OR "acquired immuno-deficiency syndrome" OR AIDS):ti,ab,kw

#1 AND #2

***ProQuest Dissertations & Theses Global (ProQuest)***

111 records - July 10, 2026

Advanced Search

Command Line Search

S1

title(insomnia? OR ((difficult* OR problem? OR trouble?) N/3 (sleep* OR asleep)) OR (disorder* N/3 sleep) OR (disturbance? N/1 sleep) OR (disrupt* N/0 sleep) OR (early N/1 (awakening OR waking)) OR ((waking OR awakening) N/3 night*) OR (fragment* N/0 sleep) OR "poor* sleep" OR "sleep complaint?" OR sleeplessness OR nightmare? OR "sleep initiation" OR (quality N/1 sleep) OR "sleep hygiene" OR "sleep restriction" OR "sleep education") OR abstract(insomnia? OR ((difficult* OR problem? OR trouble?) N/3 (sleep* OR asleep)) OR (disorder* N/3 sleep) OR (disturbance? N/1 sleep) OR (disrupt* N/0 sleep) OR (early N/1 (awakening OR waking)) OR ((waking OR awakening) N/3 night*) OR (fragment* N/0 sleep) OR "poor* sleep" OR "sleep complaint?" OR sleeplessness OR nightmare? OR "sleep initiation" OR (quality N/1 sleep) OR "sleep hygiene" OR "sleep restriction" OR "sleep education") OR subject(insomnia? OR ((difficult* OR problem? OR trouble?) N/3 (sleep* OR asleep)) OR (disorder* N/3 sleep) OR (disturbance? N/1 sleep) OR (disrupt* N/0 sleep) OR (early N/1 (awakening OR waking)) OR ((waking OR awakening) N/3 night*) OR (fragment* N/0 sleep) OR "poor* sleep" OR "sleep complaint?" OR sleeplessness OR nightmare? OR "sleep initiation" OR (quality N/1 sleep) OR "sleep hygiene" OR "sleep restriction" OR "sleep education")

S2

title("human immunodeficiency virus" OR HIV OR "acquired immune deficiency syndrome" OR "acquired immunodeficiency syndrome" OR "acquired immuno-deficiency syndrome" OR "AIDS") OR abstract("human immunodeficiency virus" OR HIV OR "acquired immune deficiency syndrome" OR "acquired immunodeficiency syndrome" OR "acquired immuno-deficiency syndrome" OR "AIDS") OR subject("human immunodeficiency virus" OR HIV OR "acquired immune deficiency syndrome" OR "acquired immunodeficiency syndrome" OR "acquired immuno-deficiency syndrome" OR "AIDS")

S1 AND S2
