# Supplementary material for: Pharmacological and Nonpharmacological Interventions to Address Insomnia Symptoms and Poor Sleep Quality Among People With HIV: Protocol for a Scoping Review
Source: JMIR Res Protoc. 2026 Oct 14;15:e98894. doi: 10.2196/98894 (PMC13664994; doi:10.2196/98894)
Supplement: Supplementary file 2 [file resprot-v15-e98894-s002.docx]

**Table 2: Domains and Subdomains Reflected in Data Extraction Tool for Scoping Review Examining Pharmacological and Non-Pharmacological Interventions to Improve Insomnia Symptoms among PWH**

| **Major Data Domain** | **Data Sub-domains** |
| --- | --- |
| *Publication Details* | First Author Last Name  Year of Publication  Journal  Country/Region of Study  Study Setting |
| *Participant Information* | Total Sample Size  Biological Sex (% female)  Mean Age  Race/Ethnicity  Average Years Living with HIV  % Virally Suppressed  CD4 Count (Mean or Range)  % on Antiretroviral Therapy (ART)  How Insomnia/Poor Sleep Was Identified (screening tool, clinical dx, self-report)  Eligibility Criteria Summary |
| *Study Design* | Study Design  Control/Comparison Condition  Randomization (Y/N)  Blinding  Study Duration/Follow-up Timepoints  % of Intervention Completers |
| *Intervention Design* | Intervention Type  Intervention Name  Intervention Description  Delivery Format  Deliver/Implementer  Dose (# Sessions/Frequency)  Intervention Fidelity (if available)  Duration of Intervention  HIV-tailored?  Theory/Framework Guiding Intervention  Medication Name (if pharmacological)  Dose/Route (if pharmacological)  Mechanism of Action (if pharmacological) |
| *Sleep-Related Outcomes* | Primary Sleep Outcome(s)  Sleep Outcome(s) Measures  Objective v. Self-Report Measures  Number of Measurement Timepoints  Primary Sleep Outcome Result (direction + significance)  Secondary Sleep Outcome(s) |
| *Additional Outcomes* | Mental Health/Substance Use Outcomes Reported  ART Outcomes Reported  Quality of Life/HrQOL Outcomes Reported  Adverse Events/Side Effects Reported |
| *Key Findings and Notes* | Summary of Key Findings  Limitations Noted by Authors |
